# Enhanced N-Butanol Sensing Performance of Cr-Doped CeO_2_ Nanomaterials

**DOI:** 10.3390/s25041208

**Published:** 2025-02-16

**Authors:** Yanping Chen, Haoyang Xu, Jing Ren, Guangfeng Zhang, Yonghui Jia

**Affiliations:** School of Science, Shandong Jianzhu University, Jinan 250100, China; yanping_c@sdjzu.edu.cn (Y.C.); 2022125105@stu.sdjzu.edu.cn (H.X.); 2022125103@stu.sdjzu.edu.cn (G.Z.); 2023125018@stu.sdjzu.edu.cn (Y.J.)

**Keywords:** gas sensing, CeO_2_, n-butanol, oxygen vacancies

## Abstract

The Cr-doped CeO_2_ nanomaterials were prepared by a simple hydrothermal method. Morphological analysis revealed that Cr doping altered the morphology and size of the CeO_2_ particles. Gas sensing tests results showed that Cr/Ce-2 has the highest response (*R_a_*/*R_g_* = 15.6 @ 10 ppm), which was 12.58 times higher than that of the pure CeO_2_ sensor. Furthermore, the optimal operating temperature was reduced from 210 °C to 170 °C. The Cr/Ce-2 sensor also displayed outstanding repeatability and gas selectivity. The improved gas sensing performance of the Cr-doped CeO_2_ sensor can be attributed to its smaller grain size and higher porosity compared to pure CeO_2_. In addition, oxygen vacancies played a pivotal role in improving the gas-sensing performance. The present work provides a new CeO_2_-based gas-sensitive material for the detection of n-butanol.

## 1. Introduction

With the development of science and technology, environmental pollution has become increasingly serious. The toxic gases emitted by industry can cause severe paralysis and irritation to humans [1]. These toxic gases are not only harmful to human health but also have an impact on safety production. Therefore, it is crucial to develop effective gas sensors that can accurately identify gas concentrations.

Metal Oxide Semiconductor (MOS) gas sensors, primarily categorized into n-type and p-type, have gained extensive applications in detecting polluting, flammable, and explosive gases, as well as toxic substances, owing to their benefits including cost-effectiveness, sensitivity, ease of operation, and robust stability [2]. CeO_2_, a typical n-type MOS, has gained considerable attention owing to its redox properties [3], chemical stability [4], high catalytic activity [5], and outstanding gas-sensing capabilities [6,7,8]. The utilization of CeO_2_ in gas sensing has been reported in the literature. Li et al. [9] fabricated three kinds of porous CeO_2_ nanosheets, each subjected to distinct annealing durations (0.5, 1, and 2 min) for the purpose of detecting CO in an ambient environment. Notably, the CeO_2_ nanosheets annealed for 2 min demonstrated remarkable reproducibility and selectivity towards CO gas. At a temperature of 450 °C, the sensor exhibited a swift response and recovery time (2 s/2 s) when exposed to 500 μL/L CO. Lai et al. [10] developed CeO_2_ nanoparticle-based micro-electromechanical system gas sensors for the detection of H_2_, NH_3_, CO, and NO_2_. The results revealed that at an optimal operating temperature of 300 °C, the sensor exhibited higher sensitivity to H_2_ (*R_a_*/*R_g_* = 0.9 @ 50 ppm) compared to other gases.

However, a significant limitation of most CeO_2_ gas sensors is their requirement for operation above 300 °C and their relatively long response–recovery times, which hinder their application in high-temperature and high-pressure environments. Conversely, CeO_2_ synthesized by metal doping can solve these challenges. Doping has been shown to be an effective strategy for modulating the electronic structure and surface activity of materials. And the incorporation of metal oxides into semiconductors is the most common method of modulating the sensitivity, selectivity, and stability of chemoresistive gas sensors [11,12]. Ahmad et al. [13] utilized Zn-doped CeO_2_ for the detection of nitroaniline gas, demonstrating favorable selectivity and remarkable sensitivity to the gas. Specifically, at a temperature of 200 °C, the response to 100 ppm of nitroaniline was recorded as 18.6 (*S* = *R_a_*/*R_g_*), with a linear response observed across a broad range of nitroaniline concentrations. Additionally, Aboud et al. [14] prepared Gd_2_O_3_/CeO_2_ composite material for CO_2_ detection and doping with Gd reduced the particle size of CeO_2_. This led to a significant improvement in sensitivity, achieving 45% (*S =* (*R_g_* − *R_a_*)/*R_a_*) for 800 ppm CO_2_ at 250 °C, compared to pure CeO_2_ (10%). Furthermore, Zakaria et al. [15] employed a Zr and V co-doped CeO_2_/TiO_2_ core–shell gas sensor for ethanol sensing. Their results showed an increase in sensitivity from 26.50 to 277.50 when using a 1% molar ratio of doped Zr^4+^ and V^3+^ for 420 ppm ethanol at 29 °C (45% RH), compared to undoped CeO_2_/TiO_2_ core/shell nanostructures. Notably, the sensor maintained stable sensitivity over an 11-month period, indicating excellent long-term stability. In addition, Cr doping can enhance the gas sensitivity of compounds. Li et al. [16] successfully synthesized Cr-doped NiO nanoparticles through a hydrothermal method specifically for benzyl mercaptan detection. Sensors doped with 25 at% Cr demonstrated the optimal response to benzyl mercaptan gas, achieving a ratio of *R_g_*/*R_a_* = 10.6 at 100 ppb. Additionally, the sensor exhibited remarkable stability over ten months of repeated measurements. In another study, Hemalatha and colleagues [17] synthesized Cr-doped ZrO_2_ for ammonia detection. Their findings revealed that the sensor with 15 at% Cr doping exhibited the highest gas sensitivity, reaching 58% (calculated as *S* (%) = (*σ_g_* − *σ_a_*)/*σ_a_* × 100%), where *σ_a_* and *σ_g_* represent the conductivity of the sensor in air and the test gas, respectively.

To our knowledge, there are few reports on the utilization of Cr-doped CeO_2_ composite materials as gas sensors. In this work, Cr-doped CeO_2_ composites were synthesized using a hydrothermal method. By thoroughly analyzing the structure, composition, and gas-sensitive properties of the synthesized materials, we discovered that the 1 mol% Cr-doped CeO_2_ composites exhibited superior performance compared to pure CeO_2_ for n-butanol detection. Specifically, they demonstrated a higher response (*R_a_*/*R_g_* = 15.6 at 10 ppm), a lower operating temperature (170 °C), and a faster response time (11 s). Consequently, Cr-doped CeO_2_ emerges as a promising material for n-butanol sensing applications.

## 2. Materials and Methods

### 2.1. Materials

Cerium nitrate hexahydrate (Ce(NO_3_)_3_·6H_2_O), chromium nitrate hydrate (Cr(NO_3_)_3_·9H_2_O), polyvinylpyrrolidone (PVP), ethanol, methanol, 2-methylimidazole, etc., were all purchased from China National Pharmaceutical Group Chemical Reagent Co., Ltd. (Beijing, China), with none of them undergoing any further purification.

### 2.2. Samples Preparation

**Figure 1:**
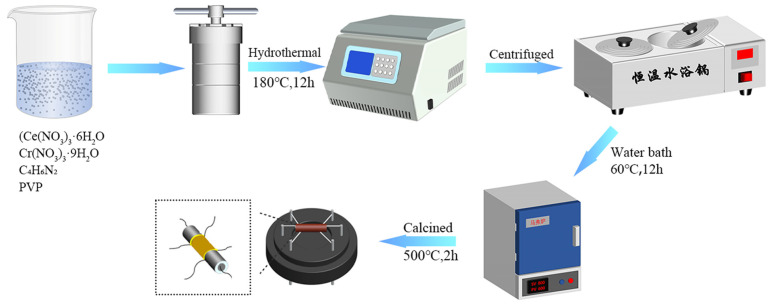
Synthesis of Cr-doped CeO_2_ nanomaterials and schematic diagram of sensor preparation.

CeO_2_-based nanoparticles with different Cr/Ce molar ratios were prepared by hydrothermal process. As shown in Figure 1, (Ce(NO_3_)_3_·6H_2_O) and Cr(NO_3_)_3_·9H_2_O were dissolved in 60 mL of ethanol at specific Ce: Cr molar ratios (100:0, 100:0.5, 100:1, 100:2) to obtain a clear solution A. Next, 1.232 g of 2-methylimidazole, 2.85 g of PVP were accurately weighed and dissolved in a 60 mL mixture of ethanol and deionized water (DIW) (in a 2:1 ratio) to obtain solution B. Solution B was then slowly poured into solution A while vigorously stirring, followed by 5 min of ultrasonication to create a homogeneous mixture. This mixture was transferred to a Teflon-lined stainless steel autoclave and heated at 180 °C for 12 h. After the reaction, the precipitate was collected, washed several times with deionized water, and dried in a water bath using thermostatic water bath at 60 °C for 12 h. Finally, the material was calcined in an air atmosphere in the muffle furnace at 500 °C for 2 h, with a calcination rate of 5 °C/min, to obtain the final product. The four products were named Cr/Ce-0, Cr/Ce-1, Cr/Ce-2, and Cr/Ce-3, respectively.

### 2.3. Characterization

The samples were indexed by an X-ray powder diffractometer (XRD, Rigaku, D8 Advance, Tokyo, Japan) with Cu-Kα radiation in the range of 10~90°. The morphology of these materials was analyzed through scanning electron microscopy (SEM, Hitachi, Regulus 8220, Tokyo, Japan). Furthermore, the size and crystal type of the materials were investigated using transmission electron microscopy (TEM, JEOL, F200, Tokyo, Japan), high resolution transmission electron microscopy (HRTEM, JEOL, F200, Tokyo, Japan), and selective area electron diffraction (SAED, JEOL, F200, Tokyo, Janan).

### 2.4. Gas Sensor Fabrication and Test

The prepared powder samples were placed in an agate mortar, and DIW was added. Then, we ground the mixture for 5 min to form a uniform paste and applied the resulting paste onto a ceramic substrate to form a tubular sensor. The ceramic tube was 4 mm long, with an external diameter of 1.2 mm, a pair of gold electrodes and four platinum wires on the surface, and a nickel-chromium heating wire passing through the inner part to regulate the sensor’s operating temperature. As shown in Figure 1, the ceramic tube was fused to the sensor base to finalize the sensor unit. The sensor was placed on a TS-60 (Weisheng Electronics Technology Co., Ltd., zhengzhou, China) aging table for 24 h. The gas-sensitive performance of the sensor was tested using a WS-30A testing device (also manufactured by Weisheng Electronics Technology Co., Ltd., zhengzhou, China), with the entire experimental process conducted through the static test method. Based on the concentration of the gas to be measured and the ideal gas equation of state “*PV* = *nRT*”, we determined the amount of substance of the gas to be measured. Then, based on the amount of substance, we determined the volume of the liquid analyte. *P* is the pressure, *V* is the volume of the gas (the volume of the gas chamber multiplied by the concentration of the gas), *n* is the amount of substance in the gas, *R* is the molar gas constant, and *T* is the temperature. Using a microliter sampler, the water of the desired concentration was fed into the closed gas chamber, rapidly evaporated into water vapor and diffused by the evaporator, and the desired relative humidity was determined by the change in the hygrometer. After the testing room was covered and ventilated, a certain volume of testing fluid was injected through the opening at the back of the experimental chamber. The evaporator turns the liquid droplets into gas, which is then combined with air, causing a change in the sensor’s resistance. Once the stabilized value was achieved, the test chamber was opened, and upon re-exposure to air, the sensor’s resistance returned to its pre-gas injection level. The software analyzed the continuous resistance change in the material under test to obtain a response–recovery curve. The response of n-type semiconductor sensor was defined as *S* = *R_a_*/*R_g_*, where *R_a_* is the sensor’s resistance in air and *R_g_* is the sensor’s resistance in the tested gas [18]. The time needed for the sensor’s overall resistance to change by 90% either after the gas is removed or after it has been exposed to the target gas is known as the response and recovery times.

## 3. Results and Discussion

### 3.1. Material Structure and Morphology

**Figure 2:**
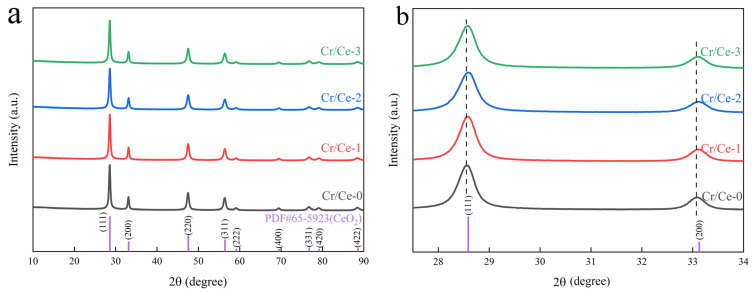
(**a**) XRD patterns of four samples; (**b**) Comparison of (111) and (200) peaks from XRD patterns.

Figure 2a shows the XRD spectra of the prepared samples, and the results show that all samples are well-crystallized and have identical diffraction peak positions and peak shapes. The diffraction peaks correspond to the (111), (200), (220), (311), (222), (400), (331), (420), and (422) crystal planes of CeO_2_ (PDF#65-5923), indicating good crystallization of the prepared sample. Due to the minimal doping of Cr, no peaks related to Cr were observed in the XRD pattern. According to Scherrer’s formula *D* = 0.89*λ*/(*β*cos *θ*), where *λ* is the X-ray wavelength, *β* is the half-peak width, and *θ* is the Bragg diffraction angle, the average crystallite sizes of Cr/Ce-0 to Cr/Ce-3 were about 19.6 nm, 18.0 nm, 16.7 nm, and 16.3 nm, respectively. The grain size decreases gradually with increasing chromium doping concentration. As shown in Figure 2b, the (111) peak is slightly shifted to a higher angle with increasing Cr concentration. The shift corresponds to a lattice distortion. This may be due to the difference in ionic radii between Ce^3+^ and Cr^3+^, where Cr^3+^ (0.61 Å) is smaller than Ce^3+^ (1.03 Å), and the smaller ionic radius is replaced by a larger one, suggesting that Cr^3+^ can be successfully doped into the CeO_2_ lattice. This is conducive to improving the gas-sensitive properties of the material [19,20].

**Figure 3:**
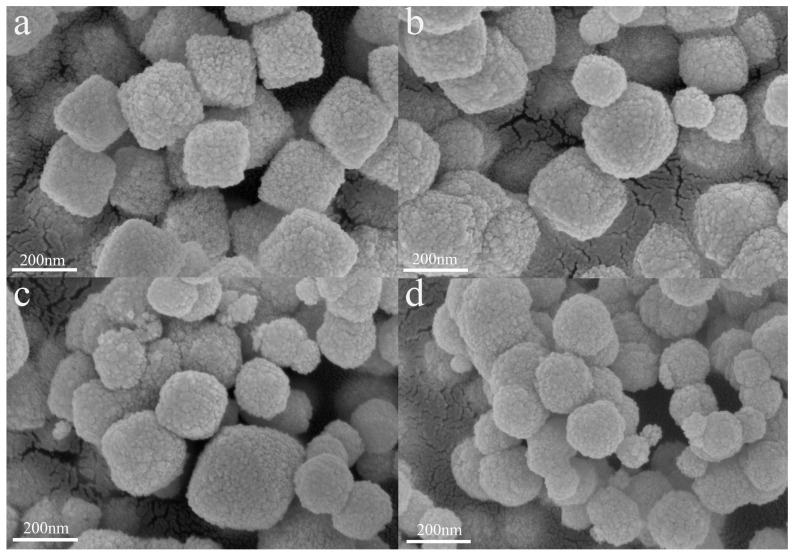
The SEM images of (**a**) Cr/Ce-0; (**b**) Cr/Ce-1; (**c**) Cr/Ce-2; and (**d**) Cr/Ce-3.

Figure 3 shows the SEM of Cr-doped CeO_2_ composites with different concentrations. The Cr/Ce-0 shown in Figure 3a is uniformly stacked by many cubic blocks with sizes of about 180–200 nm. Figure 3b–d show the completely different morphologies of Cr/Ce-1 to Cr/Ce-3 samples. As the concentration of Cr doping increased, the size of the nanocubes gradually became smaller and the morphology gradually changed to spherical. As shown in Figure 3d, the size of the nano microspheres was about 50–200 nm. The SEM size is not consistent with those obtained from X-ray diffraction (XRD) analysis, attributed to the fact that the SEM cubes or spheres are being stacked by individual nanoparticles. Among them, Figure 3c shows reduced particle size and increased pores relative to Figure 3b, while Figure 3d displays significant agglomeration and reduced pores relative to Figure 3c. Overall, the particles in Figure 3c are smaller and more dispersed, resulting in more pores and an increase in the surface area to volume ratio, ultimately enhancing the material’s gas sensing capabilities.

**Figure 4:**
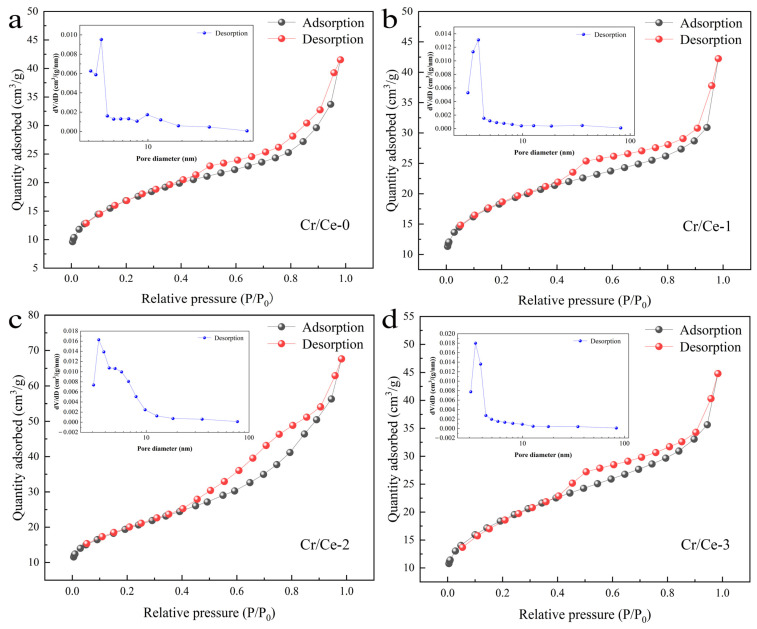
Preparation of samples (**a**–**d**) N_2_ adsorption–desorption isotherms of Cr/Ce-0 to Cr/Ce-3 and corresponding pore size distribution curves (inset).

**Figure 5:**
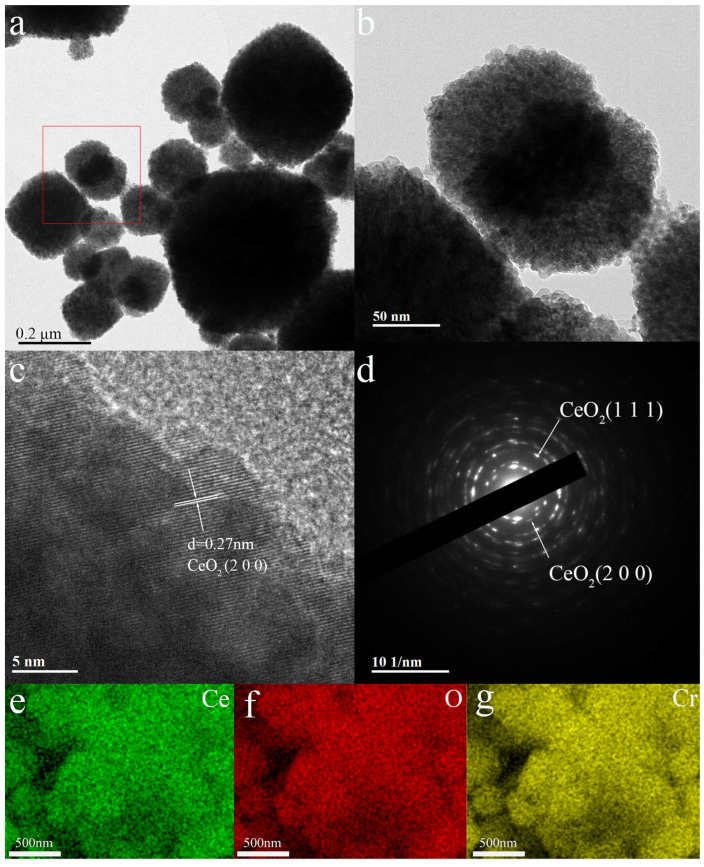
Images of Cr/Ce-2 (**a**), (**b**) TEM; (**c**) HRTEM; (**d**) SAED and (**e**–**g**) EDS.

The specific surface area and porosity of Cr-doped CeO_2_ and pure CeO_2_ were analyzed using N_2_ adsorption desorption isotherms. As illustrated in Figure 4, the BET specific surface areas of Cr/Ce-0, Cr/Ce-1, Cr/Ce-2, and Cr/Ce-3 are 59.53 m^2^/g, 65.414 m^2^/g, 69.174 m^2^/g, and 61.59 m^2^/g, respectively. By comparing these values, it is evident that Cr-doped CeO_2_ has a larger specific surface area compared to pure CeO_2_. The inset of Figure 5 shows the pore size distributions of the four materials. Upon calculation, the peaks of the pore size distributions are centered around 3.871 nm (Cr/Ce-0), 3.885 nm (Cr/Ce-1), 3.423 nm (Cr/Ce-2), and 3.555 nm (Cr/Ce-3), indicating that the pore size range of the samples are mainly distributed in the mesoporous range. The pore size distributions of Cr/Ce-0, Cr/Ce-1, Cr/Ce-2, and Cr/Ce-3 had porosities of 15.378 m^2^g^−1^nm^−1^, 16.837 m^2^g^−1^nm^−1^, 20.038 m^2^g^−1^nm^−1^, and 17.324 m^2^g^−1^nm^−1^, respectively. Notably, Cr/Ce-2 exhibits the highest porosity, offering more adsorption sites and, consequently, demonstrating superior gas-sensing performance.

Figure 5a present the TEM images of Cr/Ce-2, revealing the presence of nanoparticles of varying sizes on its surface, which are consistent with the particle sizes observed in the SEM images. Figure 5b shows an enlarged portion of the red box of Figure 5a. Because the principles of representation methods are different, it is evident that the particle size determined by XRD is substantially smaller. In particular, SEM and TEM give the real particle size, whereas XRD measures the coherent scattering size (CSD). The CSD is typically less than the particle size seen by SEM or TEM for multi-domain particles [21]. Figure 5c shows the HRTEM image of Cr/Ce-2, where lattice streaks of CeO_2_ are clearly visible. The structure with a lattice spacing of 0.27 nm is the (200) crystalline surface of CeO_2_. Additionally, Figure 5d displays the SAED image of Cr/Ce-2, revealing diffraction rings that signify the polycrystalline nature of the sample. The diffraction rings from the inside to the outside correspond to the (111) and (200) crystal planes of CeO_2_. Due to the low concentration of Cr doping, no diffraction rings or lattice fringes related to Cr were found in this image. Furthermore, Figure 5e–g exhibit the EDS spectra of Cr/Ce-2 of the three elements Ce, O, and Cr. The figures indicate a uniform distribution of these elements within the material. The presence of Ce, O, and Cr within the nanostructure of the microspheres confirms that Cr has been successfully doped into CeO_2_.

**Figure 6:**
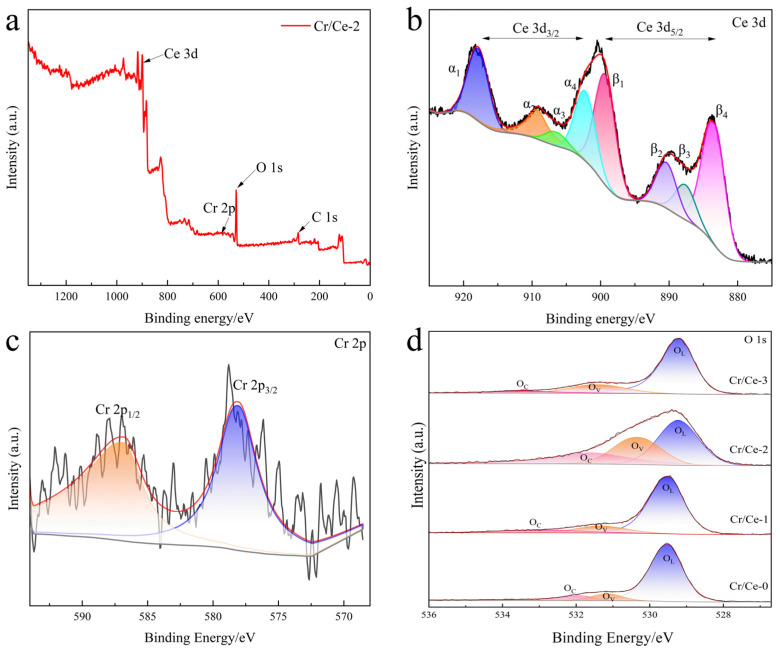
(**a**) XPS survey scan of Cr/Ce-2.; (**b**) Ce 3d spectrum of Cr/Ce-2; (**c**) Cr 2p spectrum of Cr/Ce-2; (**d**) O 1s spectrum of four materials.

The elemental composition and chemical state of the prepared samples were analyzed by XPS, with the results presented in Figure 6. From the full XPS spectrum of Cr/Ce-2 in Figure 6a, it can be seen that the prepared composite mainly consists of three elements, namely Ce, Cr, and O, which also verifies the successful doping of Cr into CeO_2_. The black lines in Figure 6b–d represent the raw data, the red lines are the fitted data, and the gray lines are the baselines. The different colored areas indicate the peaks after fitting the atoms of different valence states of each element. The Ce 3d fine spectrums of Cr/Ce-2 are shown in Figure 6b, and it can be seen that there are eight peaks in the region, which are labeled with α and β. Among them, four characteristic peaks, α_1_, α_2_, α_3_, and α_4,_ are matched with the Ce 3d_3/2_ energy level, and four characteristic peaks, β_1_, β_2_, β_3_, β_4,_ match the Ce 3d_5/2_ energy level [22,23]. According to Schubert’s study [24], these eight characteristic peaks belong to two different Ce valence states, α_1_, α_2_, α_4_, β_1_, β_2,_ and β_4_ are attributed to the Ce^4+^ valence state, while α_3_ and β_3_ are attributed to the Ce^3+^ valence state. The presence of Ce^3+^ indicates a higher number of redox active sites in Cr/Ce-2, which facilitates surface reactions. The Cr 2p fine spectrum of Cr/Ce-2 is shown in Figure 6c, and the characteristic peaks with binding energies of 577.15 eV and 586.95 eV correspond to the Cr 2p_3/2_ and Cr 2p_1/2_ energy levels, thus determining the valence state of the dopant as Cr^3+^ [25]. The introduction of Cr^3+^ increases the electron density on the surface of CeO_2_ and decreases the binding energy, and the doping leads to a charge imbalance between Ce^3+^ and Ce^4+^, generating more defective sites favorable for oxygen surface adsorption, thus enhancing the gas-sensitive performance. The O 1s fine spectra of the four materials are shown in Figure 6d, each containing three fitted peaks representing lattice oxygen (O_L_), vacancy oxygen (O_V_), and adsorbed oxygen (O_C_). Table 1 outlines the relative percentages of O_V_. Notably, Cr doping increases the percentage of oxygen vacancies on the material’s surface compared to pure CeO_2_, with Cr/Ce-2 exhibiting the highest percentage of O_V_. The XPS image confirms that, when doped into the CeO_2_ lattice, Cr^3+^ ions replace Ce^3+^/Ce^4+^ ions, creating a large number of oxygen vacancies. Oxygen vacancies are closely linked to gas-sensitive performance, as they are more prone to capturing electrons and effectively separating electron–hole pairs, thereby improving sensing performance [26,27]. Oxygen vacancies are closely linked to gas-sensitive performance, as they are more prone to capturing electrons and effectively separating electron–hole pairs, thereby improving sensing performance [26,27].

**Figure 7:**
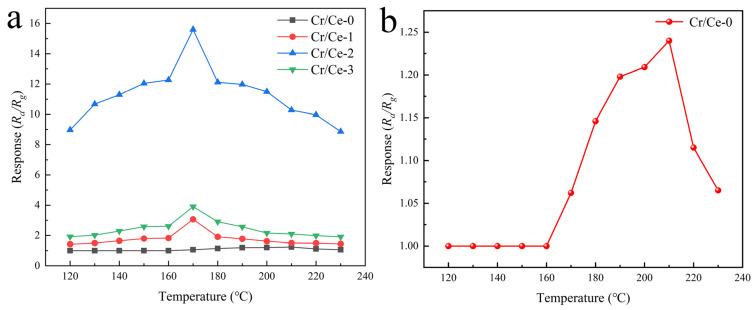
(**a**) The responses of Cr/Ce-0, Cr/Ce-1, Cr/Ce-2, and Cr/Ce-3 gas sensors as a function of the operating temperature to 10 ppm n-butanol; (**b**) Cr/Ce-0 amplified response plot.

As can be seen in Figure 7a, the gas-sensitive response of four sensors to 10 ppm n-butanol was tested within a temperature range of 120 °C to 230 °C. For the Cr/Ce-1, Cr/Ce-2, and Cr/Ce-3 samples, the response to n-butanol increases and then decreases as the operating temperature increases, with the optimum response value occurring at 170 °C. Figure 7b shows the response of the pure CeO_2_ sensor, revealing an optimum operating temperature of 210 °C. This suggests that Cr doping effectively lowers the activation energy required for the reaction between n-butanol molecules and the material’s surface. Among the materials prepared in this experiment, Cr/Ce-2 showed the highest response, achieving a value of 15.6 to 10 ppm n-butanol at 170 °C. In contrast, the responses of Cr/Ce-1 and Cr/Ce-3 to 10 ppm n-butanol at 170 °C were 3.1 and 4, respectively. At the optimal operating temperature, the balance between adsorption and desorption rates, coupled with an appropriate activation energy, maximizes the sensor’s response. However, excessively high temperatures can cause premature desorption of n-butanol molecules from the material’s surface, interrupting their interaction with oxygen molecules and significantly diminishing the response [28,29].

**Figure 8:**
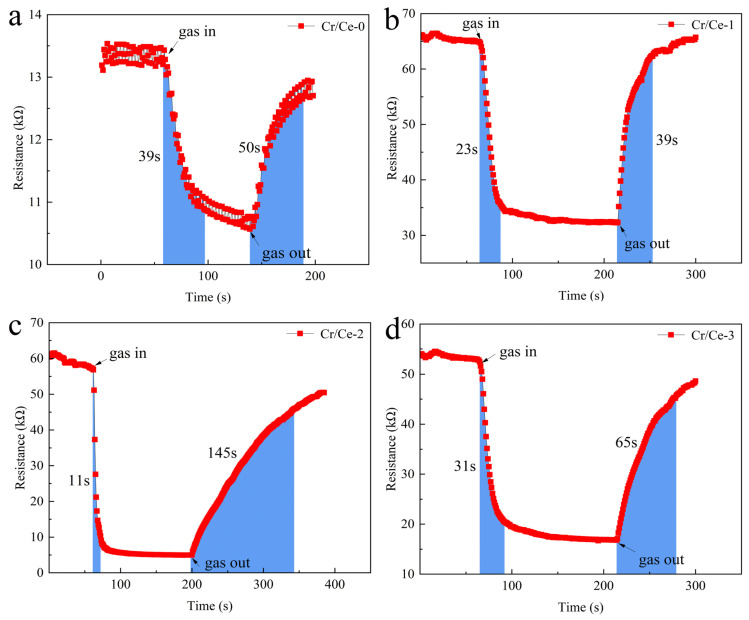
Response–recovery curve of (**a**) Cr/Ce-0; (**b**) Cr/Ce-1; (**c**) Cr/Ce-2; (**d**) Cr/Ce-3 to 10 ppm n-butanol at optimum operating temperature.

The response–recovery curves of each gas sensor at their optimal working temperature in response to 10 ppm n-butanol are displayed in Figure 8. The blue area on the left of Figure 8a–d indicates the time required for the sensor to respond, and the blue area on the right indicates the time required for recovery. As can be seen from Figure 8, when n-butanol is injected, the resistances of the four materials all show a decreasing trend. When restored to the air environment, the resistance values all show an increasing trend, and eventually recover to the initial resistance baseline position, showing typical sensing characteristics of n-type semiconductor materials. The substitution of Ce^3+^ by Cr^3+^ creates a charge imbalance, as Cr^3+^ has a different charge state compared to Ce^3+^. To compensate for this imbalance, oxygen vacancies are formed, which further contribute to the electron depletion layer on the material’s surface. This layer increases the material’s resistance in air, but when exposed to reducing gases like n-butanol, the resistance decreases due to the release of trapped electrons. The resistance of the Cr-doped sensor in air (R_a_) is higher than that of the pure CeO_2_ sensor at the same operating temperature. This may be due to the fact that Cr doping reduces the width of the charge conduction channels and the cross-sectional area available for charge conduction in CeO_2_, so more electrons are involved in the transfer during gas adsorption and desorption, leading to an increase in resistance.

In terms of response–recovery, Table 2 presents the response and recovery times for the four materials. It can be seen that Cr/Ce-2 exhibits the shortest response time. This rapid response may be attributed to the porous structure and abundant oxygen vacancies of the synthetic materials, which enhance oxygen adsorption and accelerate the surface reaction rate. Conversely, the relatively slow recovery time of Cr/Ce-2 may be due to the excessive adsorption of n-butanol gas on the surface of the material, requiring a longer period to desorb, thereby prolonging the recovery time [30].

**Figure 9:**
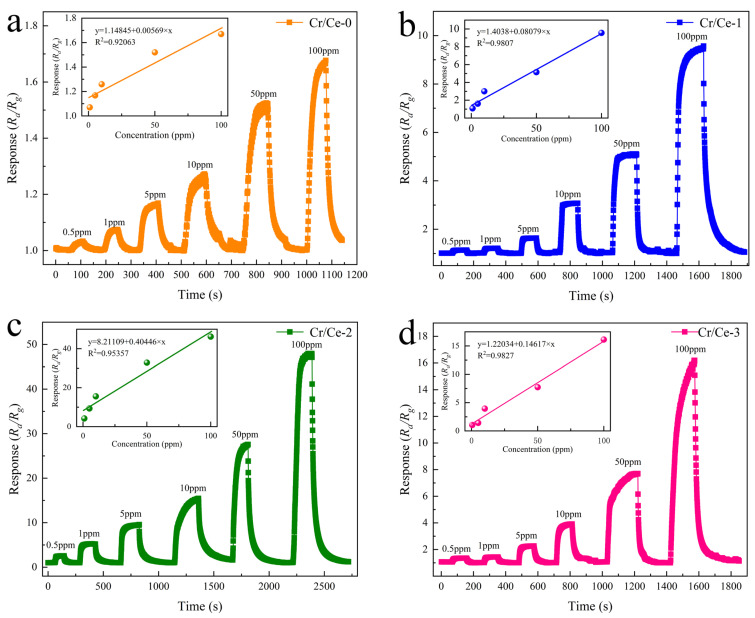
Dynamic response–recovery curves of (**a**) Cr/Ce-0; (**b**) Cr/Ce-1; (**c**) Cr/Ce-2; (**d**) Cr/Ce-3 for 0.5–100 ppm n-butanol at optimal operating temperature.

The dynamic response image of the four sensors to 0.5–100 ppm n-butanol at their ideal operating temperature is displayed in Figure 9. The dots in the inset show the response values of the individual sensors for different gas concentrations. As the concentration of n-butanol increases, the response also becomes larger. The inset shows the corresponding dynamic fitting curves, with the linear fitting curve for the Cr/Ce-2 sensor given by the formula y = 0.40446x + 8.21109, these data show a correlation coefficient of R^2^ = 0.95375, indicating a strong linear relationship between the sensor’s response and the concentration of n-butanol gas [31]. The gas concentration in the environment can be approximated based on the sensor’s response. By comparing the reactions of four sensors to n-butanol concentrations (10 ppm, 50 ppm, and 100 ppm) at their ideal operating temperatures, Cr/Ce-2 showed the highest response (15.6, 32.83, 46.21) at the three n-butanol concentrations.

**Figure 10:**
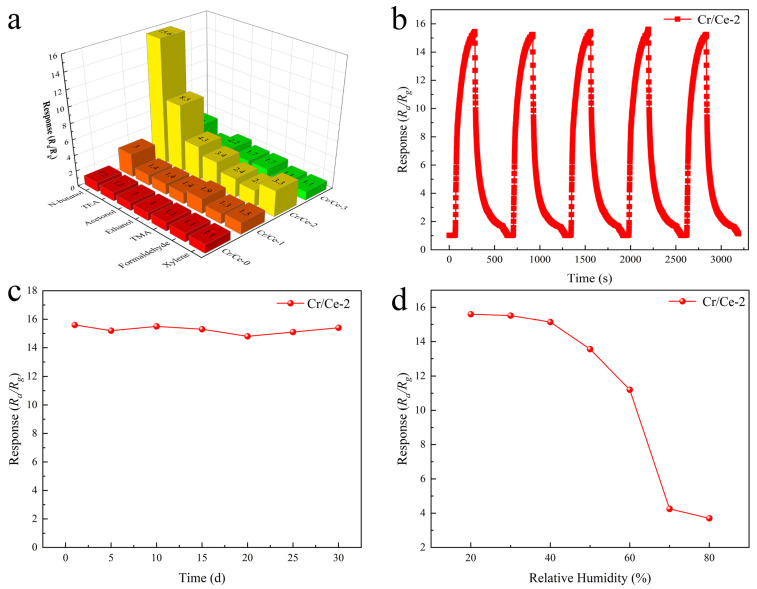
(**a**) Response of four sensors to seven gases at optimum operating temperature; (**b**) Response of Cr/Ce-2 to 10 ppm n-butanol in five consecutive cycling cycles; (**c**) Stability of Cr/Ce-2 over 30 days of testing; (**d**) Effect of relative humidity on the response of Cr/Ce-2.

Selectivity is a very significant performance parameter for gas sensors. Figure 10a shows the response of the four sensors to 10 ppm of n-butanol, triethylamine, acetone, ethanol, trimethylamine, formaldehyde, and xylene, all measured at their optimum operating temperatures. Notably, the Cr/Ce-2 sensor exhibits a response value of 15.6 to 10 ppm n-butanol, which is five to fifteen times higher than that of other gases at the same concentration. Consequently, the Cr/Ce-2 sensor demonstrates superior selectivity towards n-butanol. The repeatability and stability of gas sensors are also essential for evaluating the performance of gas sensors [32]. The results of the Cr/Ce-2 sensor tested with 10 ppm n-butanol over five consecutive cycle periods are shown in Figure 10b. The results revealed that the response values for five consecutive tests showed almost no significant change and could be recovered to their initial resistance without baseline drift. Therefore, the Cr/Ce-2 sensor has good repeatability. Furthermore, the Cr/Ce-2 sensor exhibits good long-term stability, as seen in Figure 10c, which shows a stable response to 10 ppm n-butanol over the course of a 30-day test cycle. Humidity also plays a pivotal role in gas sensor research. Figure 10d displays the Cr/Ce-2 sensor’s reaction to 10 ppm n-butanol under varying relative humidity conditions. The sensor’s response steadily decreases as relative humidity increases, with a significant drop occurring above 60% relative humidity. This phenomenon can be attributed to competition between oxygen and water molecules in the air for adsorption sites on the sensor’s surface, leading to decreased adsorbed oxygen and reduced sensor responsiveness [33]. Therefore, the prepared sensors are suitable for the environment with a relative humidity below 60%.

To evaluate the gas-sensitive performance of the Cr-doped CeO_2_ sensor, we conducted a comparative analysis with the other reported literature [34,35,36,37,38,39,40]. As shown in Table 3, we have developed a Cr/Ce-2 sensor with a higher response and faster response time than most other reported materials. In addition, from the previous characterization images, we can see that we synthesized materials with special morphology. Therefore, the Cr/Ce-2 sensor has novelty as well as great potential for development in the detection of n-butanol.

### 3.2. Gas Sensing Performances

**Figure 11:**
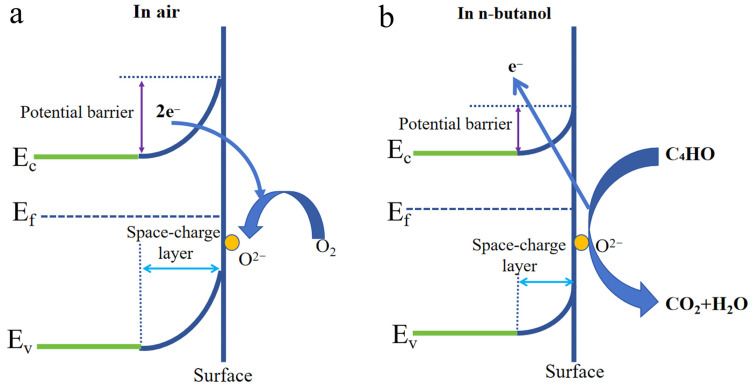
(**a**) Schematic diagram of the gas-sensitive mechanism of Cr-doped CeO_2_ in air; (**b**) Schematic diagram of the gas-sensitive mechanism of Cr-doped CeO_2_ in n-butanol.

CeO_2_, an n-type semiconductor material, has a distinct resistance value when exposed to air and target gas environments, enabling its gas detection capabilities. When oxygen molecules adhere to the surface of the material and seize free electrons, adsorbed oxygen is formed, a process that occurs naturally when the material is exposed to air. The formation of oxygen ions (O_2_^−^, O^−^, and O^2−^) is represented by Equations (1)–(3). This process leads to an increase in the material’s electrical resistance due to the depletion of electrons, resulting in the creation of an electron depletion layer on its surface [41]. When the CeO_2_ sensor is placed in an n-butanol environment (Equation (4)), n-butanol reacts with the oxygen ions generated on the material’s surface. This reaction reduces the thickness of the electron depletion layer and the grain boundary barriers, as illustrated in Figure 11a,b. Notably, the Cr-doped sensor exhibits a higher response compared to pure CeO_2_. The concentration and type of gas can be determined based on the resistance changes in the prepared sensor in different gases [42].

(1) $$O_{2}\;(\mathrm{gas})\;\to \;O_{2}\;(\mathrm{ads}).$$

(2) $$O_{2}\;(\mathrm{ads})+e^{-}\;\to \;{O_{2}}^{-}\;(\mathrm{ads}).$$

(3) $${O_{2}}^{-}\;(\mathrm{ads})+e^{-}\;\to \;2O^{-}\;(\mathrm{ads}).$$

(4) $$C_{4}H_{10}O+12O^{-}\;\to \;4{\mathrm{CO}}_{2}+5H_{2}O+12e^{-}.$$

The Cr/Ce-2 material exhibits a superior gas-sensing performance for n-butanol compared to other doped materials, possibly attributed to the following reasons: (1) The Cr-doped CeO_2_ material has a smaller grain size, and it can be seen that Cr/Ce-2 is formed by the aggregation of nanoparticles of different sizes by XRD, SEM, and BET. The different sizes of nanospheres enhance the porosity of the sensing material. The small particle size increases the number of active surface sites, thus enhancing the response. Additionally, the increased porosity provides more gas diffusion pathways, further augmenting the response [43]. (2) Oxygen vacancies are also an important factor affecting gas sensing performance. High-temperature annealing leads to oxygen segregation within the lattice, causing oxygen loss and the generation of oxygen vacancies. Moreover, Cr doping introduces additional oxygen vacancies, and the following is the flawed reaction equation following the addition of Cr^3+^:

(5) $${\mathrm{Cr}}_{2}O_{3\;\vec{{\mathrm{CeO}}_{2}}}{2\mathrm{Cr}}_{\mathrm{Ce}}^{\prime }+{3O}_{O}^{x}+V_{O}^{\cdot \cdot },$$

where we adopt the Kröger–Vink notation for trapping: ${\mathrm{Cr}}_{\mathrm{Ce}}^{\prime }$ is a Cr-substituted Ce site with three negative charges, and $V_{O}^{\cdot \cdot }$ is an oxygen vacancy that possesses two positive charges. Through electrostatic interactions, the positively charged oxygen vacancies will take electrons from the conduction band and serve as adsorption sites for the oxygen ions’ surface chemisorption, which will take up further electrons from the conduction band. Even a tiny amount of electron release can result in a somewhat large resistance shift when a reducing gas is added. In gas sensor testing, Cr doping facilitates rapid electron transport. Furthermore, a conductive process is implied by the discharge of electrons from the sensor surface, which lowers the activation energy of the sensing reaction and enhances the material’s gas-sensitive qualities [42,43,44,45].

## 4. Conclusions

In summary, a straightforward hydrothermal process was used to create Cr-doped CeO_2_ nanoparticle composites. The doping of Cr not only reduced the optimal operating temperature but also significantly enhanced the CeO_2_ sensor’s responsiveness to n-butanol detection. The improved sensing performance is mainly attributed to an increase in porosity and a higher concentration of oxygen vacancies. Specifically, the Cr/Ce-2 sensor demonstrated a response of 15.6, which is 12.58 times greater than that of the pure CeO_2_ sensor (1.24) when exposed to 10 ppm of n-butanol at the optimal temperature of 150 °C. Additionally, the sensor exhibits strong gas selectivity, repeatability, and good potential for the detection of n-butanol gas.

## Figures and Tables

**Table 1:** Relative percentages of O_V_ in four materials.

Material	O_V_
Cr/Ce-0	11.38%
Cr/Ce-1	17.59%
Cr/Ce-2	30.17%
Cr/Ce-3	12.66%

**Table 2:** Response–recovery times for four materials.

Material	Response Time (s)	Recovery Times (s)
Cr/Ce-0	39	50
Cr/Ce-1	23	39
Cr/Ce-2	11	145
Cr/Ce-3	31	65

**Table 3:** Performance comparison of various gas sensors for n-butanol.

Materials	Working Temperature (°C)	Concentration (ppm)	Response (R_a_/R_g_)	Response/ Recovery (s)	Refs.
CeO_2_-SnO_2_	110	10	10.2	-/-	[34]
Ag_2_O/CeO_2_ modified ZnO	160	10	28.9	-/-	[35]
Gd-CeO_2_	580	200	59.2	-/-	[36]
CeO_2_/Co_3_O_4_	190	10	6.54	23/7	[37]
CeO_2_/WO_3_	RT	10	7.56	-/-	[38]
Al-CeO_2_	RT	25	2.47	64/52	[39]
CeO_2_/TiO_2_	300	300	5.44	20/20	[40]
Cr/Ce-2	170	10	15.6	11/145	This work

## Data Availability

The data of our study are available upon request.
